# *Plasmodium* 18S rRNA of intravenously administered sporozoites does not persist in peripheral blood

**DOI:** 10.1186/s12936-018-2422-2

**Published:** 2018-07-27

**Authors:** Sean C. Murphy, Andrew S. Ishizuka, Zachary P. Billman, Tayla M. Olsen, Annette M. Seilie, Ming Chang, Nahum Smith, Vorada Chuenchob, Sumana Chakravarty, B. Kim Lee Sim, Stefan H. I. Kappe, Stephen L. Hoffman, Robert A. Seder

**Affiliations:** 1 Departments of Laboratory Medicine and Microbiology, University of Washington, 750 Republican St., E630, Seattle, WA 98109 USA; 2 Center for Emerging and Re-emerging Infectious Diseases, University of Washington, 750 Republican St., Seattle, WA 98109 USA; 3 Vaccine Research Center, National Institute of Allergy and Infectious Diseases, National Institutes of Health, Building 40, Room 3512, 40 Convent Drive, Bethesda, MD 20814 USA; 4 Center for Infectious Disease Research, 307 Westlake Ave N #500, Seattle, WA 98109 USA; 5 Sanaria, Inc., 9800 Medical Center Drive, Suite A209, Rockville, MD 20850 USA

**Keywords:** 18S rRNA, SSU, Small subunit rRNA, *Plasmodium*, Molecular diagnostic

## Abstract

**Background:**

*Plasmodium* 18S rRNA is a biomarker used to monitor blood-stage infections in malaria clinical trials. *Plasmodium* sporozoites also express this biomarker, and there is conflicting evidence about how long sporozoite-derived 18S rRNA persists in peripheral blood. If present in blood for an extended timeframe, sporozoite-derived 18S rRNA could complicate use as a blood-stage biomarker.

**Methods:**

Blood samples from *Plasmodium yoelii* infected mice were tested for *Plasmodium* 18S rRNA and their coding genes (rDNA) using sensitive quantitative reverse transcription PCR and quantitative PCR assays, respectively. Blood and tissues from *Plasmodium falciparum* sporozoite (PfSPZ)-infected rhesus macaques were similarly tested.

**Results:**

In mice, when *P. yoelii* sporozoite inoculation and blood collection were performed at the same site (tail vein), low level rDNA positivity persisted for 2 days post-infection. Compared to intact parasites with high rRNA-to-rDNA ratios, this low level positivity was accompanied by no increase in rRNA-to-rDNA, indicating detection of residual, non-viable parasite rDNA. When *P. yoelii* sporozoites were administered via the retro-orbital vein and blood sampled by cardiac puncture, neither *P. yoelii* 18S rRNA nor rDNA were detected 24 h post-infection. Similarly, there was no *P. falciparum* 18S rRNA detected in blood of rhesus macaques 3 days after intravenous injection with extremely high doses of PfSPZ. *Plasmodium* 18S rRNA in the rhesus livers increased by approximately 101-fold from 3 to 6 days post infection, indicating liver-stage proliferation.

**Conclusions:**

Beyond the first few hours after injection, sporozoite-derived *Plasmodium* 18S rRNA was not detected in peripheral blood. Diagnostics based on 18S rRNA are unlikely to be confounded by sporozoite inocula in human clinical trials.

## Background

*Plasmodium* parasites are the causative agents of human malaria. Infection begins when female *Anopheles* mosquitoes take a blood meal. During feeding, mosquitoes transmit sporozoite-stage parasites into the dermis. Sporozoites make their way to blood vessels and then to the liver, where they develop over the next several days. The sporozoite and liver stage parasites are clinically silent. At the completion of the liver stage, parasites are released into the bloodstream and invade erythrocytes. The resulting cyclical infection of erythrocytes is responsible for all clinical disease. During the erythrocyte stage, parasites can be detected in whole blood using several diagnostic tests such as microscopy of Giemsa-stained blood smears, lateral flow rapid diagnostic tests for parasite antigens and nucleic acid tests (NATs). In general, NATs are more analytically sensitive than other modalities [1]. The most common NAT targets are DNA genes encoding the *Plasmodium* 18S ribosomal RNAs (hereafter called 18S rDNA) or the 18S rRNAs themselves, with testing by polymerase chain reaction (PCR) or reverse transcription PCR (RT-PCR), respectively [1].

*Plasmodium* 18S rRNA/rDNA-targeted NATs are intended to detect blood-stage parasites, but they also can detect the same sequences in sporozoite and liver stages. Most evidence suggests that sporozoites transit from the mosquito inoculation site to the liver in less than an hour [2]. However, if sporozoite-derived nucleic acids, or even sporozoites, continued to circulate for days following sporozoite exposure, these nucleic acids could generate false positive results for NATs intended to monitor for erythrocyte infection. An earlier study reported this type of NAT positivity in mice [3]. Abkallo and colleagues reported that *Plasmodium yoelii* 18S rDNA was detectable in peripheral mouse blood by qPCR after *P. yoelii* sporozoite injection, but before emergence of infected erythrocytes from the liver at about 48 h. Compared to *Plasmodium falciparum*, *P. yoelii* has a shorter liver stage with erythrocyte stage emergence ~ 48 h post-inoculation. In the Abkallo study, CBA mice were infected with 2.5 × 10^4^ *P. yoelii* sporozoites by tail vein injection, and blood was sampled by capillary action from the tail vein at later time points. For 90 min following *P. yoelii* sporozoite injection, 18S rDNA was detected in tail vein peripheral blood at low and decreasing concentrations. 18S rRNA was next detected ~ 24 h post-injection (at ~ 50–100 copies of 18S rDNA/μL of blood) followed by a decrease to baseline and then a marked rise at 48 h corresponding with erythrocyte stage infection and blood smear positivity. Based on these data, the authors concluded that NAT positivity prior to the emergence of erythrocyte stage parasites was from circulating pre-erythrocytic parasites. Such a result could complicate the use of *Plasmodium* 18S rRNA/rDNA NATs to assess infection in pre-clinical and clinical trials when using attenuated sporozoite vaccines that must be monitored for safety or using wild-type sporozoites for challenge studies [4]. Thus, to further investigate whether pre-erythrocytic parasites are a confounder of peripheral blood NATs, additional experiments using the 18S rRNA biomarker were conducted in mice and non-human primates (NHP).

## Methods

### Mice and *Plasmodium yoelii* infections

Female BALB/cj mice (4–6 weeks old) were obtained from Jackson Laboratories (Barr Harbor, ME), housed in an IACUC-approved animal facility and used under an IACUC-approved protocol. Wild-type *P. yoelii* 17XNL sporozoites were obtained by salivary gland dissection from *Anopheles stephensi* mosquitoes reared at the Center for Infectious Disease Research (CID Research, Seattle, WA). *Plasmodium yoelii* sporozoites were injected intravenously in a 100–150 μL volume via tail vein injection or by retro-orbital injection as noted in “Results” section. Parasites were purified using the accudenz gradient method [5] with minor modifications as reported [6]. Dried blood spots were collected by tail vein bleeds from alive mice (5–10 μL per spot) using a site in the distal tail usually 1–2 cm from the injection site. Venous whole blood was collected by cardiac puncture in euthanized mice as noted in the Results section. Venous whole blood samples were preserved in NucliSENS lysis buffer (bioMérieux) immediately after collection using a ratio of 50 μL of blood to 2 mL of lysis buffer. DBS from tail vein collections were dried and desiccated. All preserved samples were stored at − 80 °C until extraction.

### Non-human primate and *Plasmodium falciparum* infections

Wild-type PfSPZ were freshly dissected and purified at Sanaria, Inc. (Rockville, MD) and transported for 20 min to the IACUC-inspected NHP facility. Four rhesus macaques were intravenously infected with 6.5 × 10^6^ PfSPZ under an IACUC-approved NHP protocol. Three or 6 days later, the animals were humanely euthanized and liver, lung, spleen and EDTA-anticoagulated whole blood samples were collected. Tissues were snap frozen in liquid nitrogen (250 mg) and whole blood was immediately preserved in lysis buffer using a ratio of 50 μL of blood to 2 mL of lysis buffer. All preserved samples were stored at − 80 °C until extraction; frozen samples were shipped on dry ice. At the time of extraction, snap frozen tissues were emulsified in lysis buffer by bead beating using a ratio of 250 mg of tissue to 10 mL of lysis buffer. Tissues/blood were also obtained from uninfected animals (called ‘pre-inoculation’).

### Nucleic acid extraction

Total nucleic acids were extracted from mouse whole blood (50 μL) and mouse dried blood spots (~ 5–10 μL) on an EasyMag instrument (bioMérieux) and eluted in a volume of 53 μL as described [7]. At the time of processing, DBS were laser-cut as reported [8]. NHP blood (50 μL) and tissues (derived from 50 mg tissue equivalents from emulsified lysates of 50 mg or larger 250 mg samples, see Results) samples were extracted on an Abbott m2000sp and eluted in a volume of 53 μL as described [8].

### qRT-PCR and qPCR

**Fig. 1:**
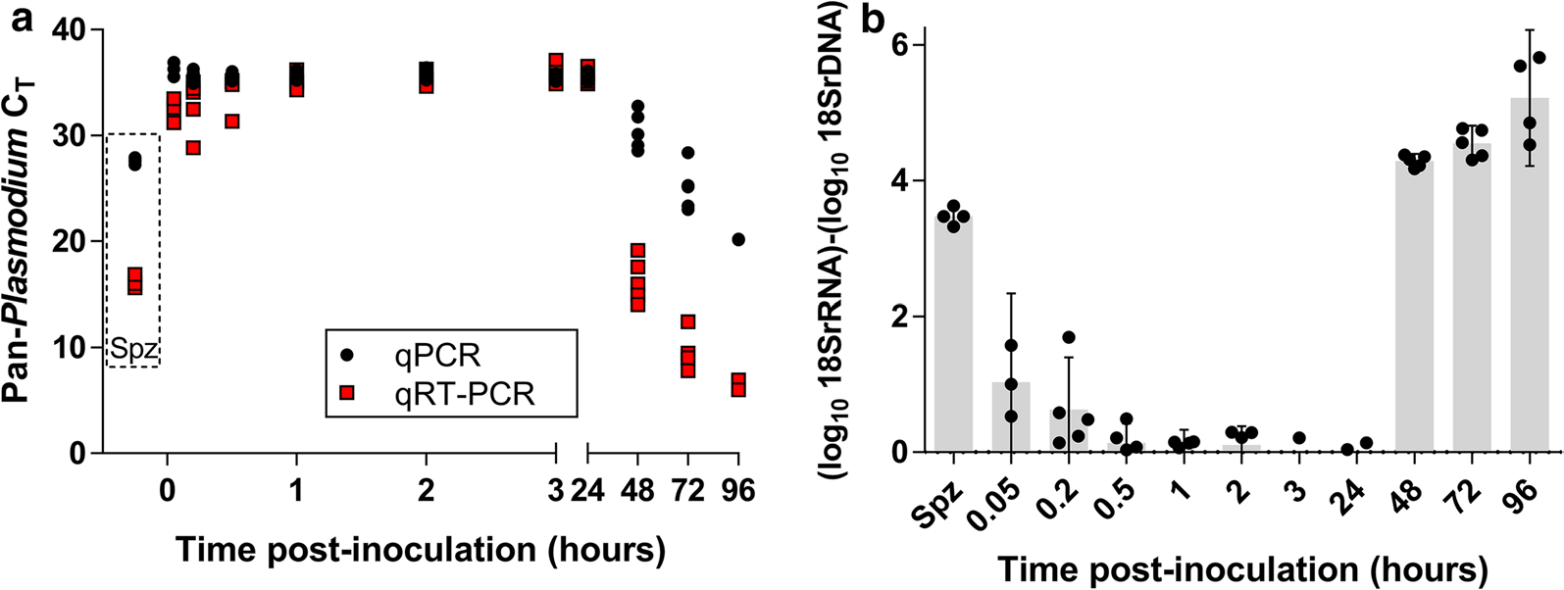
*Plasmodium yoelii* 18S rRNA versus 18S rDNA in blood collected from the tail vein of BALB/cj mice after tail vein inoculation of sporozoites. **a** *Plasmodium yoelii* sporozoites were injected by the tail vein and then whole blood was collected onto dried blood spots at the indicated hour post-inoculation, preserved in lysis buffer and extracted for total nucleic acids. qPCR or qRT-PCR was performed and the C_T_s are plotted. Red squares: qRT-PCR (18S rDNA + 18SrRNA), Black circles: qPCR (18S rDNA). Dashed box indicates 5 × 10^4^ freshly-dissected sporozoites (Spz). Each data point represents a unique blood sample obtained from an individual animal at the stated time point. **b** Using the standard curve of diluted parasites, the log_10_ copies/mL difference between 18S rRNA and 18S rDNA was calculated for each time point for each mouse

Quantitative reverse transcription PCR (qRT-PCR) for *P. yoelii* 18S rRNA was performed using primers/probes and conditions as reported [6] using 5 μL of template. qRT-PCR for *P. falciparum* was performed using primers, probes and conditions as reported [8] on an Abbott m2000rt using 15 μL of template or BioRad CFX1000 using 5 μL of template. With the exception of the DNA:RNA assay reported in Fig. 1, qRT-PCR results were calibrated against an absolute RNA standard curve to determine exact copy number values. For the rRNA:rDNA experiment in Fig. 1, absolute quantification was not possible because the DBS input volume varied from 5 to 10 μL per spot—instead cycle thresholds (C_T_) were used. To analyse DNA only, the RT enzyme was omitted and quantitative PCR (qPCR) was performed; to analyse RNA, the RT enzyme was included but DNase was not used since rRNA is 1000–10,000 times more abundant than rDNA in intact parasites, depending on the assay and parasite species [7, 8]. In mouse blood samples, detection of 18S rRNA was defined as a two-fold or greater increase in 18S rRNA versus 18S rDNA.

## Results

### *Plasmodium yoelii* 18S rRNA/rDNA detected when injection and sampling are both via tail vein reflects non-viable, residual parasite 18S rDNA

To mimic the aforementioned Abkallo study, BALB/cj mice were injected in the tail vein with 1 × 10^4^ *P. yoelii* sporozoites and were repeatedly sampled from the tail vein thereafter. RT-PCR and PCR generated positive albeit late C_T_s from tail vein samples for all samples collected thereafter (Fig. 1a). However, even immediately after injection, the log_10_ difference in 18S rRNA versus 18S rDNA was much less than is measured in intact sporozoites. Like infected erythrocytes, intact *P. yoelii* sporozoites express much higher 18S rRNA concentrations than their coding rDNAs (> 1000-fold higher; Fig. 1b, spz bar). In contrast, the difference in 18S rRNA versus 18S rDNA in samples post-*P. yoelii* sporozoite injection was only tenfold higher immediately after injection, suggesting an increased contribution of 18S rDNA. Although absolute quantification was not performed for this experiment, the estimated number of sporozoites detected per uL of mouse blood during these timepoints based on the laboratory’s experience is estimated to be no more than 1–2 spz per uL (S. Murphy, pers. commun.). By 1 h post-inoculation, there was no difference in RT-PCR versus PCR C_T_s and the total amount of nucleic acid was much less than that required to constitute a single intact sporozoite per sample. Thus, the results indicate that only residual 18S rDNA was detected beyond 1 h post-injection (Fig. 1 and Table 1). In this study, positive detection of 18S rRNA was qualitatively defined as twofold or greater increase over 18S rDNA for a given sample. With this definition, there was no 18S rRNA detection beyond 30 min after injection. The high C_T_s measured when samples were taken later than 30 min post-administration corresponded to quantities of 18S rRNA/rDNA nucleic acids that would not be adequate to constitute a single intact parasite [7, 8]. As expected, at the start of the mouse erythrocytic stage, the difference between 18S rRNA versus rDNA increased to > 10,000-fold, consistent with the start of erythrocyte stage infection and a high per-parasite 18S rRNA content for intact, viable parasites. These data demonstrate that detection of *P. yoelii* 18S rDNA can occur in mice when *P. yoelii* sporozoites are inoculated via the tail vein and blood samples are likewise obtained from the tail vein.

**Table 1:** Summary of experiments to address route-specific *Plasmodium* 18S rRNA positivity in mouse studies

Mouse studies	18S rRNA (# positive)^a^	18S rRNA C_T_ (range)	18S rDNA (# positive)	18S rDNA C_T_ (range)	ΔC_T_^b^ (range)
Tail vein inoculation/tail vein sampling experiment					
Immediate	3/3	31.21–33.48	3/3	35.56–36.93	2.08 to 5.73
0.2 h	3/5	28.84–35.09	5/5	34.91–36.30	0.81 to 6.18
0.5 h	1/5	31.35–35.22	5/5	35.14–36.05	0.14 to 3.79
1 h	0/5	34.33–36.20	5/5	35.22–36.20	− 0.99 to 1.03
24 h	0/5	34.87–36.55	5/5	35.10–36.13	− 0.42 to 0.84
48 h	5/5	14.05–19.16	5/5	28.56–32.80	13.64 to 14.51
Retro-orbital inoculation/cardiac puncture sampling experiment					
24 h	0/3	N/A	0/3	N/A	N/A

Tail vein (3–5 μL dried blood spots); cardiac puncture (50 μL whole blood)

*N/A* not applicable

^a^18S rRNA positivity defined as a ≥ 2-fold relative increase in 18S rRNA compared to rDNA

^b^ΔC_T_ equals the C_T_ for the qPCR (rDNA) minus the C_T_ for the qRT-PCR (rRNA) for an individual sample irrespective of positivity as described above such that a high ΔC_T_ indicates a large amount of 18S rRNA relative to the coding DNA

### Circulating *P. yoelii* 18S rRNA is not detected during pre-erythrocytic stages of infection in mice when injection and sampling sites are spatially distinct

To eliminate sample site contamination as a factor, an experiment was performed wherein the *P. yoelii* sporozoite administration site and the blood sampling site were spatially separated. BALB/cj mice were retro-orbitally injected with 2 × 10^4^ wild-type *P. yoelii* sporozoites and then euthanized 1 day later and blood was collected by cardiac puncture. No *P. yoelii* 18S rRNA was detected in 50 μL whole blood samples 24 h after sporozoite inoculation (n = 3 mice) (Table 1). All nucleic acid extractions and RT-PCR assays incorporated *P. yoelii*-infected blood positive controls and uninfected blood negative controls. These data demonstrate that *P. yoelii* 18S rRNA is not detectable in peripheral blood 24 h post-administration when the injection and sampling site are spatially distinct.

### Lack of circulating *P. falciparum* 18S rRNA in peripheral blood during pre-erythrocytic stages of infection in rhesus macaques

**Fig. 2:**
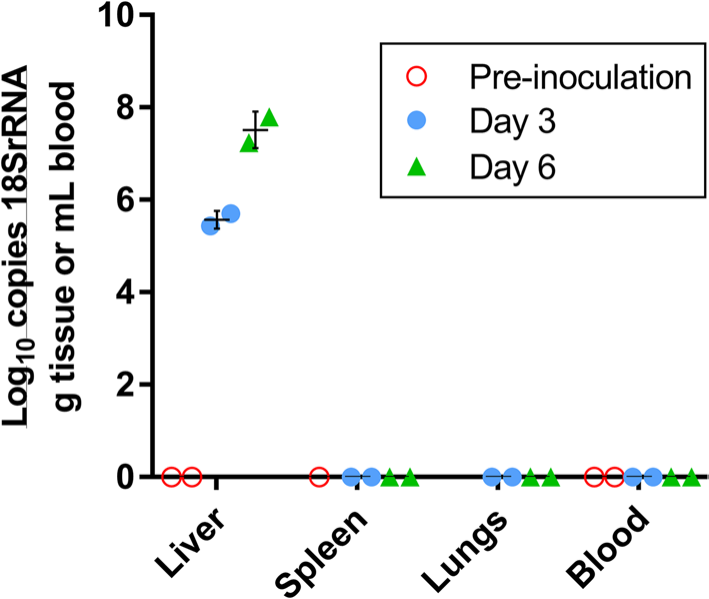
*Plasmodium falciparum* 18S rRNA biomarker in rhesus macaques before and after high-dose PfSPZ inoculation. Tissue samples and whole blood were collected and preserved in lysis buffer as described. Nucleic acids were extracted and RT-PCR performed as described. The *Plasmodium* 18S rRNA biomarker was detected only from liver samples. Open circles, pre-inoculation; closed circles, day 3 post-inoculation; closed triangles, day 6 post-inoculation. Bars show mean ± standard deviation. Spleen was tested from only one uninfected animal, and lung samples were not tested from uninfected animals. Each data point represents a unique sample obtained from an individual animal at the stated time point

To test whether *P. falciparum* 18S rRNA circulates in larger animals, a NHP study was conducted in rhesus macaques. PfSPZ invade rhesus hepatocytes but with lower efficiency than human hepatocytes [9, 10], and rhesus do not support blood stage *P. falciparum* infections [11]. Four rhesus macaques (5 kg each; ~ 250 mL circulatory volume) were intravenously infected with 6.5 × 10^6^ freshly-dissected PfSPZ on day 0. Peripheral blood was collected immediately prior to inoculation. Two animals were euthanized on each of day 3 and 6, and blood, liver, spleen and lung tissues were collected. *Plasmodium falciparum* 18S rRNA was not detected in the blood of any animal pre- (day 0) or post-inoculation (days 3 or 6) indicating that PfSPZ are cleared from the circulation within 3 days of injection (Fig. 2).

### Accumulating *P. falciparum* 18S rRNA in the liver stage of rhesus macaques

Tissue samples were also obtained from spleen, lung and liver in infected rhesus macaques. *Plasmodium falciparum* 18S rRNA was not detected in any of the lung or spleen samples three or 6 days post-infection. However, *P. falciparum* 18S rRNA was detected in liver samples from all animals after inoculation. Liver from an uninfected animal showed no such positivity. Samples for liver RT-PCR were derived from ~ 250 mg snap-frozen liver samples that were lysed and extracted for nucleic acids and subsequently tested by 18S rRNA RT-PCR [8]. *The P. falciparum* 18S rRNA liver burden of these animals was higher at day 6 post-infection compared to day 3 (Fig. 2). On day 3, the first two livers contained an average 3.9 × 10^5^ copies per gram of *P. falciparum* 18S rRNA. By day 6, the mean *Plasmodium* 18S rRNA copy number in the livers of the two remaining animals was 4.0 × 10^7^ copies per gram of liver, an 101-fold increase compared to the mean copy number from the two livers collected on day 3.

## Discussion

The *Plasmodium* 18S rRNA/rDNA biomarker is a sensitive diagnostic marker that is able to achieve earlier detection of infection compared to blood smears [7, 12]. However, conflicting evidence about the persistence of sporozoite-derived 18S rRNA led to concerns about the potential for false positive results due to persistently circulating sporozoites or their byproducts.

Here, studies in mice demonstrated that *P. yoelii* sporozoite 18S rRNA does not persistently circulate in peripheral blood and suggests that timing and concentration of the pre-erythrocytic 18S rDNA positivity 24 h post-challenge in the Abkallo study may have been due to re-sampling of locally deposited contaminating 18S rDNA. Direct data comparison between studies is limited since the assay used in the Abkallo study was a DNA-only test for *Plasmodium* 18S rRNA genes. However, in agreement with that report, in this study low amounts of *Plasmodium* 18S rRNA/rDNA could be detected. The rRNA versus rDNA comparison performed here suggests that this signal was likely from residual, locally deposited contaminating *P. yoelii* 18S rDNA. Thus, the 18S rRNA approach to parasite detection was advantageous since such tiny amounts of nucleic acid template do not constitute the 18S rRNA content of a single parasite and would have been deemed negative by 18S rRNA assays even if blood was collected from the tail vein site. In 50 μL blood samples obtained distally from the inoculation site in mice, there was no evidence of peripheral circulation of *P. yoelii* sporozoite-derived 18S rRNA 1 day after administration. It is also notable that these cardiac puncture blood samples contained 10–20 times more volume of blood per sample than the tail vein-collected dried blood spots, further supporting the conclusion that there is no long lasting, peripherally circulating *P. yoelii* 18S rRNA during the liver stage.

Studies in rhesus macaques also showed no evidence for persistent *P. falciparum* 18S rRNA circulation following administration of an exceptionally high dose of freshly-dissected PfSPZ. The PfSPZ dose given to NHP (6.5 × 10^6^) was > 2000-fold higher than the standard 3.2 × 10^3^ PfSPZ of Sanaria^®^ PfSPZ Challenge (aseptic, purified, cryopreserved PfSPZ) that always cause blood stage parasitemia in human subjects [13, 14], and comparably higher than the standard five mosquito bite dose used for controlled human malaria infections (CHMI) [15]. The NHP data support the conclusion that *P. falciparum* parasites and their 18S rRNAs do not circulate in the days following *P. falciparum* sporozoite inoculation. These data, coupled with the relatively small number of sporozoites delivered by infected mosquito bites or by PfSPZ Challenge and the intended timing of diagnostic sample collection starting on day 6 or later, make the risk of sporozoite-induced false positives negligible in human challenge studies.

In addition, the NHP studies are consistent with expansion of liver-stage parasites (roughly 101-fold from day 3 to 6 post-infection) in these animals. While immunogenicity of PfSPZ vaccines has been assessed in rhesus [16], pre-erythrocytic protection studies are usually not tested in rhesus because they do not develop blood-stage *P. falciparum* infections after receiving *P. falciparum* sporozoites [11]. However, *P. falciparum* sporozoites invade many different cell types [17] including rhesus hepatocytes in vitro (albeit with lower efficiency than human hepatocytes) [9, 10]. *Plasmodium falciparum* sporozoites that successfully invade rhesus hepatocytes in vitro subsequently express PfEXP1, a protein not expressed in sporozoites [9], indicating that the parasites continue to develop in these cells. Wild-type *P. falciparum* sporozoites in human hepatocytes proliferate ~ 30,000-fold during their 6.5 day development [2] with most proliferation occurring in the latter part of the cycle. The 101-fold increase observed from day 3 to 6 in rhesus macaques in this study demonstrates proliferation of *P. falciparum* in infected rhesus hepatocytes, though the current data does not match the proliferative potential of *P. falciparum* as measured in human liver. This study was limited by the small number of NHP (n = 2 animals per timepoint) and a relatively small number of liver tissue samples overall. More systematic liver sampling using larger biopsies collected throughout the pre-erythrocytic stage could be useful for understanding and measuring the full growth potential of the *P. falciparum* liver stage in rhesus macaques. The data also suggest that rhesus could be further investigated for use in testing pre-erythrocytic stage-targeted vaccines and/or drugs using a liver-stage *P. falciparum* 18S rRNA endpoint as a measure of efficacy.

In agreement with the mouse and NHP findings presented herein, CHMI studies also support the lack of circulating *P. falciparum* sporozoite-derived 18S rRNA after *P. falciparum* sporozoite inoculation. *Plasmodium falciparum* 18S rRNA was not detected 7, 10 or 28 days after administration of genetically-attenuated GAP3KO sporozoites delivered by 150–200 *P. falciparum* GAP3KO-infected mosquito bites [18]. The GAP3KO study is highly relevant to the question of persistent circulating 18S rRNA/rDNA since the attenuated parasite does not lead to formation of infected erythrocytes so any circulating *P. falciparum* 18S rRNA would have been presumed to be *P. falciparum* sporozoite-derived.

Like the animal studies presented herein, rising biomarker positivity from day 6 to 7 post-infection in CHMI studies at multiple centres [7, 19–23] strongly supports the well-studied timing of erythrocyte stage emergence from the human liver and suggests that testing starting on days 6–7 post-inoculation is appropriate in human clinical trials. These findings are further corroborated by earlier studies aimed at measuring the duration of the liver stage by culturing parasites during days 5–9.5 post-inoculation [24] where *P. falciparum* could only be cultured from *P. falciparum* sporozoite-infected human volunteers from day 6.5 onward. In another early study, human volunteers were bitten by infected mosquitoes and their blood was then sub-inoculated into different human recipients at timepoints thereafter [2]. *Plasmodium falciparum* infections could only be successfully sub-inoculated into recipients when donor blood was collected within 1 h of the donor’s original mosquito bites and not again until 5–6 days later when the erythrocyte stage had begun. The overall conclusion is that in the days that follow sporozoite exposure, sporozoites do not circulate in peripheral blood. Therefore, the data collectively indicate that in humans the presence of *P. falciparum* 18S rRNA days after sporozoite inoculation reflects *P. falciparum* erythrocyte stage parasite emergence, not the persistence of *P. falciparum* sporozoite-derived 18S rRNA.

## Conclusions

In sum, 18S rRNA is a sensitive marker of *Plasmodium* blood stage infection. Beyond the first few hours immediately after injection, sporozoite-derived *Plasmodium* 18S rRNA does not persist in peripheral blood. Diagnostic tests based on 18S rRNA are unlikely to be confounded by sporozoite inocula.

## Abbreviations

NHP: non-human primates
NAT: nucleic acid test
PfSPZ: *P. falciparum* sporozoite
PCR: polymerase chain reaction
qPCR: quantitative PCR
qRT-PCR: quantitative RT-PCR
RT-PCR: reverse transcription PCR
rDNA: ribosomal DNA
rRNA: ribosomal RNA
